# Differences between visual hemifields in identifying rapidly presented target stimuli: letters and digits, faces, and shapes

**DOI:** 10.3389/fpsyg.2013.00452

**Published:** 2013-07-19

**Authors:** Dariusz Asanowicz, Kamila Śmigasiewicz, Rolf Verleger

**Affiliations:** ^1^Institute of Psychology, Jagiellonian UniversityKrakow, Poland; ^2^Department of Neurology, University of LübeckLübeck, Germany

**Keywords:** RSVP, visual perception, hemispheric asymmetry, hemispheric specialization, lateralization, left visual-field advantage

## Abstract

The right hemisphere has been shown to play a dominant role in processing of visuo-spatial information. Recently, this role has been studied in the two-stream rapid serial visual presentation task. In this task, two alphanumerical targets are embedded in left and right simultaneous streams of rapidly changing letters. The second target (T2) is identified better in the left than in the right visual field. This difference has been interpreted as advantage of the right hemisphere (RH). However, a disadvantage of the left hemisphere (LH) could not be excluded so far. The LH, specialized for processing of verbal stimuli, might be overloaded due to constant input of letters from both visual fields. In the present study, this overload hypothesis was tested by reducing demands on verbal processing (Experiment 1), and by overloading the RH with non-verbal stimuli: faces (Experiment 2) and irregular shapes (Experiment 3). The left visual field advantage proved to be largely independent from the level of verbal load and from stimulus type. Therefore, although not entirely disproving the overload hypothesis, these results suggest as the most parsimonious explanation this asymmetry reflects a RH advantage, presumably in perceptual and attentional processing, rather than a LH disadvantage caused by verbal overload.

## Introduction

Spatio-temporal dynamics of visual information processing has been recently studied using a two-stream variant of the rapid serial visual presentation (RSVP) task (Shih, 2000; Holländer et al., 2005; Verleger et al., 2009, 2010, 2011; Akyürek et al., 2010; Śmigasiewicz et al., 2010). In this task, participants have to identify two consecutive targets, T1 (e.g., a red letter) and T2 (e.g., a black digit), embedded in either of two rapidly changing streams of successive distractors (e.g., black letters). The streams are presented in the left and right visual fields simultaneously, T1 is presented in the left or in the right stream, and T2 follows T1 with different lags either in the same or in the opposite stream. Identification of T1 is usually equally accurate in both streams, or slightly better in the right visual field (RVF) (Śmigasiewicz et al., 2010), which is consistent with left hemisphere (LH) specialization in processing of verbal or symbolic stimuli, like letters, words, and Arabic numbers (Dien, 2009; Dehaene and Cohen, 2011). In contrast, T2 is identified up to 30% better in the left visual field (LVF) than in the RVF (Holländer et al., 2005; Verleger et al., 2009, 2010, 2011 Śmigasiewicz et al., 2010) and is also rated to occur earlier in the LVF than in the RVF (Matthews et al., 2013). These findings are not only utterly contradictory to our subjective feeling of being equally aware of visual events in both hemifields, but also contrast with small VF effect sizes usually observed in behavioral studies of visuo-spatial processing. Typically, differences between VFs amount to around 10–20 ms in response time, or few percentage points in accuracy (see Hellige et al., 2010 for a review), and may not be easily replicable (Verfaellie et al., 1988; Evert et al., 2003; see also Hellige et al., 2010).

The mechanism underlying this prominent LVF advantage in two-stream RSVP has still remained undetermined. Although right hemisphere (RH) superiority for perceptual or attentional processes has been suggested as a possible explanation (Holländer et al., 2005; Verleger et al., 2009, 2011), this visual field asymmetry may actually result from LH disadvantage rather than from RH advantage (Hellige et al., 1979; Holländer et al., 2005; Verleger et al., 2010). In all previous two-stream RSVP studies alphanumerical verbal stimuli were used as targets and distractors, which stimuli have been shown to be processed more efficiently by the LH in most right-handed individuals (Pujol et al., 1999). According to the callosal relay model of functional hemispheric lateralization (Zaidel, 1983; Moscovitch, 1986), information that cannot be efficiently processed by one hemisphere due to lack of specialized systems is relayed to the more competent hemisphere through the corpus callosum. Imaging studies provided direct evidence for this model, showing that a left-lateralized linguistic neural network is strongly engaged by alphabetic stimuli, regardless of the input hemifield (Cohen et al., 2002). A recent electrophysiological study has shown that the transfer of verbal information from the LVF/RH to the LH begins already about 100 ms after stimulus onset, thereby suggesting that interhemispheric communication includes sharing of low level information already at early stages of processing (Doron et al., 2012). In two-stream RSVP, the rapidly presented series of distractor letters have to be processed, at least to some degree, in search for targets. Therefore, the LH, responsible for processing of verbal stimuli (Dien, 2009; Dehaene and Cohen, 2011), might have to cope with constant input from both VFs simultaneously, and thus could be overloaded (Hellige et al., 1979; Verleger et al., 2010). The overload may disrupt the LH's ability to single out the second target from the two streams of letter distractors presented in rapid succession.

Several previous studies have shown that LH efficiency may be indeed compromised by increased demands for verbal processing. For instance, Hellige and colleagues (Hellige and Cox, 1976; Hellige, 1978; Hellige et al., 1979) demonstrated that a concurrent verbal memory task, which is supposed to tax the LH, impairs identification of laterally presented stimuli more in the RVF than in the LVF, and may even lead to a LVF advantage in tasks in which usually a RVF advantage is observed. It has also been argued that the LH should be more affected by Stroop interference than the RH, due to the lateralization of language-related processes (see MacLeod, 1991). Several studies with a lateralized Stroop task have suggested that this might hold true (Schmit and Davis, 1974; Franzon and Hugdahl, 1987; Weekes and Zaidel, 1996; Gier et al., 2010), although the alternative interpretation of the asymmetry as due to RH superiority in attentional control (like the usual interpretation of the two-stream RSVP asymmetry) is also plausible (Asanowicz et al., 2012). Another piece of evidence that seems to support the overload hypothesis comes from a two-stream RSVP study, which has shown that repetitive transcranial magnetic stimulation (rTMS) applied to the left parietal cortex increased, to some extent, the LVF advantage in T2 identification, whereas rTMS to the right hemisphere did not bring about any significant changes in the asymmetry (Verleger et al., 2010). The LH, as being supposedly more engaged during the task might have been more susceptible to applied disruption.

The present study aimed to further investigate whether the overload hypothesis can explain the LVF advantage in T2 identification in two-stream RSVP. Two approaches were applied to this end. First, the verbal processing demands of two-stream RSVP were diminished by reducing the distractor load, presenting only the distractors directly preceding and following T1 and T2, and by reducing the target load, requiring participants to identify T2 only, rather than T1 and T2 (Experiment 1). In line with the overload hypothesis, the LVF advantage—expected to be observed when using the standard two-stream RSVP procedure—should decrease or even disappear with this reduced load. This is because the LH, released from processing the letter distractors or the T1, should improve target identification up to a level comparable to the RH. The second approach relied on overloading the RH by presenting stimuli whose processing is supposed to be lateralized to the RH: faces (Experiment 2) and non-verbalizable irregular shapes (Experiment 3), instead of letters and digits. In line with the overload hypothesis, the RH load should reverse the hemispheric asymmetry, and thus produce an advantage of the RVF, rather than of the LVF. Alternatively, if due to some stimulus-independent factor, the LVF advantage will still be present despite these experimental manipulations.

## Experiment 1

The aim of the first experiment was to test whether decreasing the verbal load will relieve the LH and improve T2 identification in the RVF. To this end, we reduced the load produced by distractors and the load produced by T1. In order to measure effects of the load produced by distractors, one group performed the standard version of the task with letters as distractors in the entire stream, while in the second group the number of distractor letters was reduced so that only the distractors preceding and following each target were presented. Similar variations of the number of stimuli have been used to manipulate load in various types of tasks (e.g., Hellige et al., 1979; Lavie et al., 2004). In order to reduce the load produced by identifying T1, a condition was included for both groups where T1 had to be ignored and only T2 had to be identified. This condition is also supposed to provide a baseline of visual-field asymmetry relatively unaffected by processing demands due to the requirement of identifying T1, like in Holländer et al. (2005) and many previous (one-stream) RSVP studies (see Nieuwenstein et al., 2009, for boundary conditions of T1 effects). Additionally, as in the previous two-stream RSVP experiments, the two targets were presented with different lags and counterbalanced across VFs to ensure uncertainty of T2 occurrence, to minimize potential effects of expectations and endogenous orienting of attention (Verleger et al., 2009). The overload hypothesis will be confirmed by decreased size of the LVF advantage in the conditions with fewer distractors and with one target only to identify, brought about by improved T2 identification in the RVF.

### Method

#### Participants

Forty-four right-handed undergraduate students from Jagiellonian University participated in the experiment for course credit. Twenty-two of them (14 females, 8 males) took part in the experiment with the standard two-stream RSVP stimuli. Their mean age was 19.3 years (*SD* = 1.0), and their scores in the Edinburgh Handedness Inventory (Oldfield, 1971) were 87.7 (*SD* = 11.3). The other half of the sample participated in the experiment with fewer distractor stimuli. From the originally twenty-two participants in this group, two were excluded due to very low accuracy of T1 identification (below 30%), almost approaching chance level. In the remaining sample 12 were female and 8 male, their mean age was 20.2 (*SD* = 1.7), and their scores in the Edinburgh Handedness Inventory were 86.0 (*SD* = 13.7). All participants had normal or corrected-to-normal vision, reported normal color vision, and no history of neurological disorders.

#### Stimuli, apparatus, and procedure

**Figure 1:**
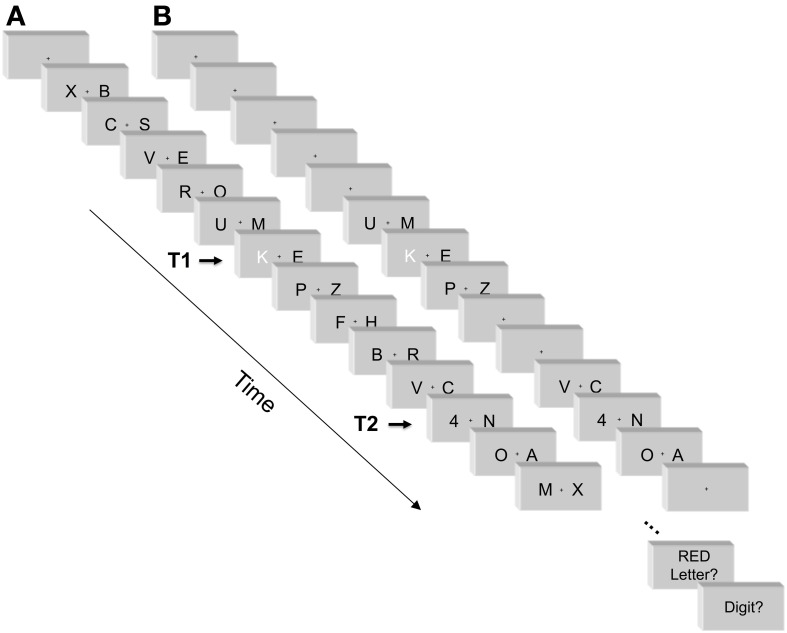
**An example of stimuli and sequence of events in a trial with standard number of distractors (A), and with reduced number of distractors (B).** See Methods for details. Red color is replaced here by white.

***Two-stream RSVP task.*** A sample stimulus sequence is illustrated in Figure 1. Two streams of black capital letters of the Latin alphabet were presented in the left and right visual field simultaneously on the white background of a 21' screen. The frame rate of the monitor was 60 Hz, i.e., frame duration equaled 16.7 ms. Each pair of stimuli was displayed for a period of seven frames, 117 ms. Subsequent letters were displayed one after another without inter-stimulus intervals. Letter font was Helvetica 35, thus letters were 11 mm high (0.8° visual angle). Their midpoints were 16 mm off screen center (1.1°), implying distances of their inner edges from screen center of around 11 mm (0.8°; varying between letters). Fixation was marked by a small dot positioned centrally on the screen (0.2° × 0.2°). In each trial, two target stimuli were displayed. The first target (T1) was a red capital letter (D, F, G, J, K, or L). The second target (T2) was a black digit (ranging from 1 to 6). The set size of six targets allows to decrease the number of correct lucky guesses, hence raises the reliability of the task. The remaining black letters displayed during the whole trial constituted the distractor set. The stimuli were presented via DMDX software (Forster and Forster, 2003).

Each trial started with a fixation period of 800 ms followed by a presentation of 12–20 subsequent pairs of stimuli. The fixation point was displayed throughout the whole trial. T1 was preceded by five, seven, or nine pairs of distractor letters, thus participants did not precisely know when it would occur. T2 followed T1 with lag 1 (no distractor letters between T1 and T2; stimulus onset asynchrony, SOA, equal to 117 ms), lag 3 (two pairs of distractors occurred between T1 and T2, SOA = 350 ms), or lag 5 (four pairs of distractors occurred between T1 and T2, SOA = 583 ms). T1 and T2 were presented in the left or right visual field with equal probability. In half of the trials, T2 occurred in the same VF as T1 ("same-side T2"), and in the other half in the opposite VF ("opposite-side T2"). Each trial ended with five letter pairs following T2. Therefore, trial length varied from 12 pairs of stimuli (when T1 came in the 6th letter pair and T1-T2 lag was 1) to 20 (when T1 came in the 10th letter pair and T1-T2 lag was 5). Target stimuli were randomly selected from the target sets. Distractor stimuli were randomly selected with replacement from the letter set, but consecutive and simultaneously presented distractors could not be identical.

Both for the group with normal number of distractors and the group with fewer distractors (2.1.2.2), there were two task conditions: dual and single-target. Both T1 and T2 had to be identified in the first condition, while T1 was ignored and only T2 had to be reported in the second condition. At the end of each trial, the fixation cross extinguished and a response screen appeared, displaying the six targets and the instruction to press the appropriate key on the computer keyboard indicating which red letter (T1) and black digit (T2) were displayed in the trial. In the single-target condition there was only the screen about T2. In the dual-target condition, the T2 response screen was preceded by the T1 response screen. Participants were informed that response times did not matter and that some responses had to be given even if the right answer was not known. The next trial started immediately after the response on T2. Participants were also instructed to keep central fixation throughout the whole trial, until the onset of the response screen. We did not record eye movements by an eye tracking device, because none was available in our Krakow lab. We had shown in previous studies that the LVF advantage in T2 identification cannot be explained by an eye movement bias, as the effect was still obtained even under strict control of fixation by means of infrared oculography (Verleger et al., 2009; Experiment 3; Verleger et al., 2013).

Varying the lag between T1 and T2 (1, 3, or 5), the side of T1 (left, right), and the side of T2 (left, right) resulted in 12 combinations of T1-T2 sequences, which were replicated 36 times (432 trials) in random order for either task condition (single and dual target). These two conditions were presented in two separate blocks, with order counterbalanced between participants. The whole experiment lasted up to one and a half hour. Both conditions were preceded by two short practice blocks, each consisting of six trials. During the first practice block, stimuli were presented in slow motion, with a display time of 500 ms, instead of 117 ms. The second practice block was performed with normal settings. During these practice trials, feedback about accuracy was given after each response.

***Two-stream RSVP task with fewer distractors.*** The task is illustrated in Figure 1. As a major change from the standard task, distractor letters occurred only directly before and after T1 and T2 (with one obvious exception at lag 1, where T2 followed T1 directly, as in the standard procedure). All other distractor letters were removed, and the fixation-cross was presented alone on the screen instead. All other aspects were identical to the standard task. In particular, the intervals between fixation point onset and T1 remained the same as in the standard procedure: 800 ms fixation period plus an interval equivalent to the five, seven, or nine letter pairs preceding T1. By this, participants did not precisely know when T1 would occur. Also, the interval between T2 offset and the end of the trial remained the same as in the standard task.

#### Data analysis

In the dual-target task, the percentage of correctly identified T1 was calculated from all trials, and the percentage of correctly identified T2 was computed from all correctly identified T1 trials. In the single-target task the percentage of correctly identified T2 was calculated from all trials. Accuracies of T1 and T2 identification in the dual-target task were analyzed separately by means of a 2 × 2 × 3 × 2 repeated measures analysis of variance (ANOVA) with Target Side (left, right; with target being T1 or T2, depending on analysis), Side Change (same side or different side of T1 and T2), and Lag (1, 3, or 5) as within-subject factors and the between-subjects factor Number of Distractors (standard number of distractors vs. reduced number of distractors). Since our interest was in VF asymmetry, effects of Side Change, Lag and Number of Distractors will be reported only if interacting with Target Side. To compare T2 identification between dual and single target tasks, a 5-way ANOVA was conducted with the additional within-group factor Task (single vs. dual-target task), focusing on moderating effects of Task on the LVF, i.e., on interactions of Task × Target Side.

### Results

**Figure 2:**
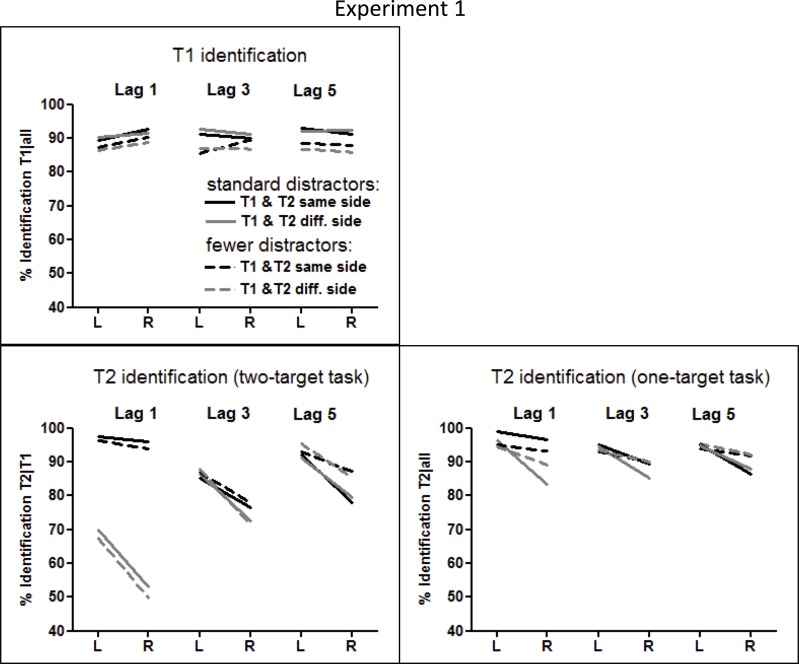
**Identification rates of T1 and T2 in Experiment 1.** Results are displayed for T1 in the **upper left panel**, for T2 from the dual-target task in the **lower left panel**, and for T2 from the single-target task in the **lower right panel**.

Mean identification rates of T1 and T2 are compiled in Table 1 and presented in Figure 2.

**Table 1:** **Percentages of correct target identification (with standard deviations) under each condition of Experiment 1**.

**Lag**	**1**				**3**				**5**			
**Side of the other target**	**Same**		**Opposite**		**Same**		**Opposite**		**Same**		**Opposite**	
**Target VF**	**Left**	**Right**	**Left**	**Right**	**Left**	**Right**	**Left**	**Right**	**Left**	**Right**	**Left**	**Right**
**T1**												
Standard procedure	89.3 (10.5)	92.6 (6.6)	90.3 (9.7)	91.4 (6.9)	91.2 (9.5)	90.0 (6.7)	92.8 (6.9)	91.3 (7.3)	92.9 (8.1)	91.3 (7.0)	92.2 (7.0)	92.4 (7.3)
Reduced number of distractors	87.4 (7.9)	90.3 (10.2)	86.4(10.2)	88.7 (9.8)	85.6 (10.7)	89.4 (9.4)	87.1 (10.5)	86.8 (12.2)	88.5 (7.8)	87.9 (8.7)	86.8 (9.8)	85.8 (13.0)
**T2/T1 DUAL TARGET TASK**												
Standard procedure	97.6 (3.0)	96.1 (3.7)	70.0 (19.6)	53.0 (18.6)	85.2 (11.2)	76.5 (15.1)	86.4 (13.1)	72.6 (19.3)	92.2 (7.8)	78.0 (13.2)	91.2 (10.7)	79.6 (16.6)
Reduced number of distractors	96.4 (5.6)	93.8 (5.2)	67.5 (17.6)	49.9 (14.6)	87.0 (10.7)	77.9 (14.3)	87.8 (9.8)	71.5 (18.1)	93.1 (7.5)	87.2 (9.8)	95.3 (7.2)	85.5 (11.4)
**T2 SINGLE TARGET TASK**												
Standard procedure	99.0 (1.6)	96.5 (3.9)	96.3 (5.5)	83.3 (11.6)	95.1 (5.7)	89.3 (6.7)	94.4 (4.7)	85.2 (12.1)	95.1 (5.7)	86.4 (8.6)	94.7 (5.5)	87.8 (13.9)
Reduced number of distractors	95.1 (11.7)	93.2 (13.7)	94.6 (19.1)	89.2 (18.3)	93.1 (17.7)	90.1 (18.5)	93.5 (18.3)	90.0 (17.5)	93.8 (19.0)	91.9 (17.6)	95.4 (16.7)	92.2 (18.3)

#### T1 identification in the dual-target task

T1 was correctly identified in 90% of trials (Figure 2, upper left panel), somewhat better with the standard number of distractors than with few ones, though not significantly so (*F*_(1, 40)_ = 2.8, *p* = 0.10, η^2^ = 0.06), and equally well in LVF and RVF (T1 Side: *F* < 1.0), except for a RVF advantage at lag 1 (T1 Side × Lag: *F*_(2, 80)_ = 6.7, *p* = 0.003, η^2^ = 0.14; T1 Side at Lag 1: *F*_(1, 40)_ = 5.3, *p* = 0.026).

#### T2 identification in the dual-target task

T2 was correctly identified in 82% of T1-correct trials (Figure 2, lower left panel). As expected, a clear-cut LVF advantage was observed (*F*_(1, 40)_ = 72.0, *p* < 0.0001, η^2^ = 0.64), modulated by Side Change and Lag, as indicated by the T2 Side × Side Change × Lag interaction (*F*_(2, 80)_ = 11.6, *p* < 0.0001, η^2^ = 0.22). When T1 and T2 were on the same side, the LVF advantage was very small at lag 1 and increased at lags 3 and 5 (T2 Side × Lag for same side T2: *F*_(2, 80)_ = 10.4, *p* < 0.0001). When T1 and T2 were on opposite sides, the LVF advantage slightly decreased from lag 1 to lag 5 (T2 Side × Lag for the opposite-side T2: *F*_(2, 80)_ = 3.7, *p* = 0.035). The LVF advantage was significant at each of the six Side Change the small 2% LVF advantage when T2 occurred at lag 1 on the same side as T1 (all *Fs*_(1, 40)_ ≥ 6.1, *p* ≤ 0.018).

Crucially, the T2 Side effect was the same for either Number of Distractors condition (main effect of Number of Distractors and interaction with T2 Side: *F* < 1.0), indicating no difference in the LVF advantage between the two conditions. Other interactions with these two factors were also not significant, except the marginally significant T2 Side × Lag × Number of Distractors interaction (*F*_(2, 80)_ = 2.7, *p* = 0.076, η^2^ = 0.06), which reflects the slightly decreased LVF advantage at Lag 5 when the number of distractors was reduced (T2 Side × Number of Distractors for Lag 5 only: *F*_(1, 40)_ = 4.0, *p* = 0.051; LVF vs. RVF at lag 5 for reduced number of distractors: *F*_(1, 19)_ = 24.6, *p* < 0.001), possibly due to a ceiling effect with left-side targets (Figure 2).

#### T2 identification in single-target vs. dual-target task

When T1 was ignored, T2 was correctly identified in 92% of all trials (lower right panel of Figure 2), 10% better than in the dual-target task (*F*_(1, 40)_ = 28.6, *p* < 0.0001, η^2^ = 0.41). These benefits from having to identify one target only were larger for RVF than for LVF (Task × T2 Side: *F*_(1, 40)_ = 16.8, *p* < 0.0001, η^2^ = 0.29). Yet there was still a LVF advantage in separate analysis of the single-target task (*F*_(1, 40)_ = 35.0, *p* < 0.0001, η^2^ = 0.46), although decreased in comparison to the dual-target task. This decrease of the LVF advantage equaled only 1% when T1 and T2 occurred on the same side, whereas when T1 and T2 were on different sides, the LVF advantage decreased about 8% (Task × T2 Side × Side Change: *F*_(1, 40)_ = 5.0, *p* = 0.031, η^2^ =0.11; Task × T2 Side for same-side T2: *F*_(1, 40)_ = 8.4, *p* = 0.006, η^2^ = 0.17; Task × T2 Side for different-side T2: *F*_(1, 40)_ = 13.0, *p* = 0.001, η^2^ =0.25). This interaction was only marginally modified by Lag (Task × T2 Side × Side Change × Lag: *F*_(2, 80)_ = 2.3, *p* = 0.10, η^2^ = 0.05), but when analyzed for each lag separately, the Task × Side Change modulation of the LVF advantage was in fact true only at lag 1 (Task × T2 Side × Side Change at lag 1: *F*_(1, 40)_ = 7.0, *p* = 0.011, at lag 3: *F*_(1, 40)_ = 2.1, *p* = 0.15, and at lag 5: *F*_(1, 40)_ < 1.0, *p* = n.s.). Thus, these complex interactions simply reflected that the small LVF advantage in the dual-target task for same-side lag-1 T1-T2 sequences, where identification rates were at ceiling, could hardly be further reduced, whereas the large LVF advantage in the other combinations of T1-T2 sequence shrank in the single-target task. This reduction of the large LVF advantage for different-side T2 in the single-target task, where the immediately preceding T1 could be ignored, was most probably related to the fact that the general difference between same-side and different-side T2 at lag 1 was reduced (but not completely abolished) from the dual-target to the single-target task. Thus, the Task × Side Change × Lag interaction amounted to *F*_(2, 80)_ = 154.2, *p* < 0.0001, η^2^ = 0.79, and the Task × Side Change interaction for lag 1 only, amounted to *F*_(1, 40)_ = 254.5, *p* < 0.0001.

Of importance, although the right panel of Figure 2 may suggest that the LVF advantage was smaller in the single-target task with fewer distractors than with the standard number of distractors, particularly with lags 1 and 3 when T1 and T2 were on different sides, no interaction of Task × Number of Distractors × T2 Side became significant (*F*'s ≤ 2.0, *p* ≥ 0.16).

### Discussion

The results of the two-stream RSVP with the standard series of distractors showed, as expected, a clear-cut LVF advantage in T2 identification, while T1 was identified equally well in both VFs, with a small trend to a RVF advantage. Thereby, previous results of studies using this task were replicated (e.g., Verleger et al., 2009; Śmigasiewicz et al., 2010; including studies where eye movements were strictly controlled by means of an eye tracker: Verleger et al., 2009; Experiment 3; Verleger et al., 2013)^1^, constituting an appropriate reference for the task with fewer distractors.

The reduction of distractors was supposed to decrease verbal load, thereby testing the assumption that the LVF is a consequence of overloading the left hemisphere with verbal input. As the experiment has shown, this manipulation had no impact on the LVF advantage, except for the marginally decreased asymmetry at lag 5 where performance generally improved with reduced number of distractors (see Figure 2). The crucial point might have been that with lag 5, in contrast to lag 1 and lag 3, stimulus streams differed between the conditions with full and with reduced number of distractors. With lag 1 and lag 3 between T1 and T2, the lags were filled by distractors equally in the full-number and the reduced-number-of-distractors conditions. But for lag 5, there was an empty interval at frames 2 and 3 after T1 in the reduced-distractors condition, followed by the onset of distractors preceding T2 at frame 4. Therefore, as soon as this empty interval occurred (at frame 2), participants could know that T2 would follow three frames (=350 ms) later, reducing temporal uncertainty. Moreover, the reappearance of distractors at frame 4 might have produced an alerting effect. The latter effect might be less relevant because a similar alerting effect is supposed to occur by the sudden onset of distractors before T1 in the reduced-distractors condition, yet T1 identification did not improve. So it was probably by the reduction of temporal uncertainty (cf. Niemi and Näätänen, 1981) that performance generally improved at lag 5 when the number of distractors was reduced. The corresponding reduction of LVF advantage in this condition may be a ceiling effect due to the higher overall accuracy at lag 5 in the task with fewer distractors, cf. Boles et al. (2008) for an extensive discussion of the dependence of measures of asymmetry on the overall performance level. It appears that with fewer distractors, the task of T2 identification at lag 5 became very similar to the task of T1 identification (thus comparably easy) due to the gap without stimulation occurring between T1 and T2 with lag 5. This seems to be confirmed by the fact that T2 at lag 5 was identified distinctly worse than T1 with the standard number of distractors (*p* = 0.001), while there was no significant difference with the reduced number of distractors (*p* = 0.11), in contrast to lag 3 and lag 1 where the difference between T1 and T2 identification was significant in both conditions. We might therefore conclude that the results of Experiment 1 suggest no relationship between verbal load produced by background letter stimuli and the LVF advantage in T2 identification under the dual-target condition, which opts against the overload hypothesis.

It may be argued, though that the reduced number of distractors still was sufficiently high to produce overload. To detail, there were still eight letters preceding T2, consisting of the pair of distractors preceding T1, of T1 and its accompanying distractor, of the pair following T1, and of the pair preceding T2. These eight letters (4 pairs × 2 sides), according to the LH overload hypothesis, would have to be processed by the LH and might have overloaded it to an extent not less than, say, when twenty letters had preceded. However, this argument does not apply at the same extent to the lag-1 condition. To detail, in this case, four letters only preceded T2 in the reduced-distractors condition, consisting of the pair of distractors preceding T1 and of T1 and its accompanying distractor. Yet also this appreciable reduction of the number of distractors did not have any moderating effect on the LVF advantage. It may still be argued that already these four preceding letters had completely overloaded the LH. However, testing this assumption by further reducing the number of distractors becomes difficult within the present paradigm because distractors preceding and trailing T1 would then have to be abolished altogether, which entails changes in overall discriminability of T1 and T2 and in general difficulty of the task. Therefore, what may be concluded is that the overload hypothesis was not confirmed as far as could be tested within the limits of the present task.

The present experiment also showed that when T1 had to be ignored, the LVF advantage decreased, as compared to the dual-target task. This reduction of VF asymmetry might be interpreted as supporting the overload hypothesis, because ignoring T1 decreases the verbal demands of the task when the LH verbal system is released from the necessity of T1 processing. On the other hand, this reduction of VF asymmetry might simply be due to a ceiling effect in the LVF. Being already high in the dual-target task, T2 identification in the LVF had much less space to improve in the single-target task compared to T2 presented in the RVF. In favor of this interpretation as a ceiling effect, the reduction of VF asymmetry closely followed improvement of T2 accuracy in the single-target task in general, being largest when T2 occurred at lag 1 in the stream different from T1. Importantly, the fact that there was still some LVF advantage present in the single-target task indicates that explicit identification of T1 is not necessary to evoke VF asymmetry in identification of the following T2 [cf. results by Nieuwenstein et al. (2009), for effects of ignoring T1 in the task with one central stream], which also suggests that this VF asymmetry is not related to the overload of the LH by target letters.

To summarize, the LVF advantage for T2 was neither abolished by reducing the number of distractors nor by letting T1 be ignored. These two results may be interpreted as converging evidence against the LH overload hypothesis. On the other hand, there were still some letters preceding T2 and possibly producing LH overload even when the number of distractors was most reduced, and the possibility of ignoring T1 did reduce (though not abolish) the LVF advantage. Therefore, these results cannot be taken as definite answer to the studied question either.

## Experiment 2A

Here, we introduced another strategy to further investigate the overload hypothesis. Instead of decreasing the load of the LH, we attempted to overload the RH. To this end, we used stimuli supposed to be preferentially processed by the RH. Lateralization in processing of human faces by the RH seems to be comparable to lateralization in processing verbal information by the LH (see Dien, 2009, for meta-analysis). Hence, according to the overload hypothesis, using images of faces as targets and distractors would result in an asymmetry reversed from using letters and digits, leading to a RVF advantage (see for similar ideas Hellige et al., 1979; Holländer et al., 2005). On the other hand, if the asymmetry occurs due to some general RH advantage, the LVF advantage will still be observed, independently of the type of stimuli.

### Method

#### Participants

Participants were recruited from the same population and fulfilled the same inclusion criteria as in Experiment 1. From the originally twenty-two participants, one person was excluded due to accuracy of T1 identification near chance level (17%). The remaining participants were 17 females and 4 males, their mean age was 19.5 (*SD* = 0.9), and their mean score in the Edinburgh Handedness Inventory was 84.0 (*SD* = 19.1).

#### Stimuli, apparatus, and procedure

**Figure 3:**
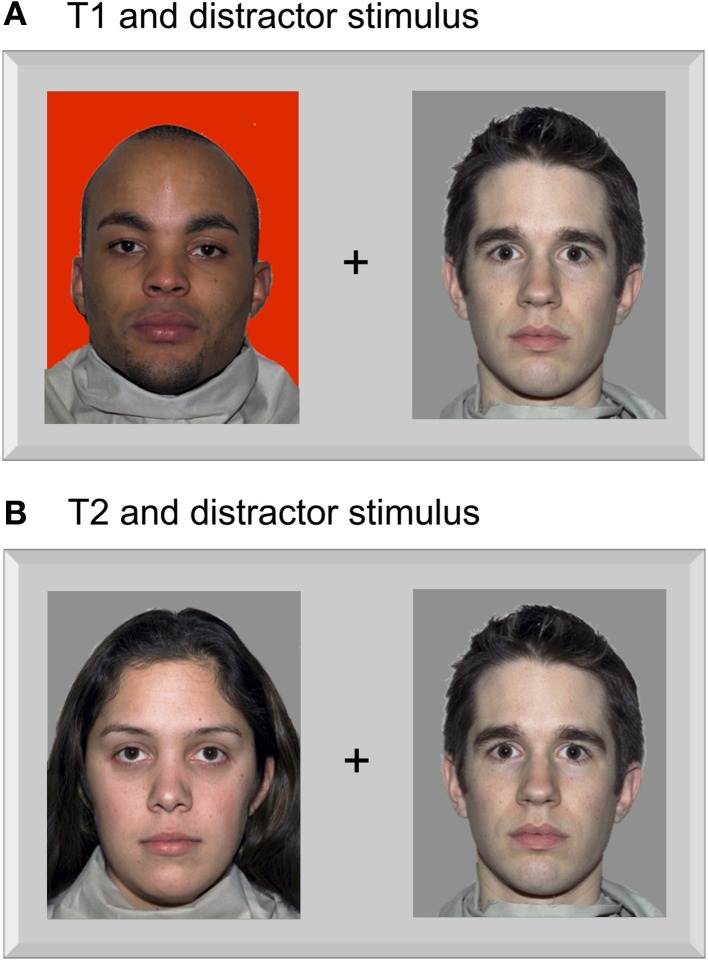
**An example of the face-stimuli used in Experiment 2 as T1 (A) and T2 (B).** Pictures were taken from the NimStim Set of Facial Expression (Tottenham et al., 2009; http://www.macbrain.org/resources.htm).

Pictures of faces were presented instead of letters and digits. Twenty-six pictures of male faces and six pictures of female faces were taken from the NimStim Set of Facial Expression (Tottenham et al., 2009; http://www.macbrain.org/resources.htm). All pictures showed emotionally neutral face expression. These stimuli were 10 mm wide and 15 mm high (0.7° × 1.0° visual angle), somewhat larger than the letters used in Experiment 1. Distance between the inner edge of pictures and the fixation point was 11 mm (0.8°). The first target (T1) was one of six pre-selected male faces displayed on a red background, in analogy to the red T1 letter in Experiment 1. The second target (T2) was one of the six female faces displayed on a white background, in analogy to the digit T2 in Experiment 1. The distractor set consisted of the remaining twenty male faces displayed on white background. All other parameters of the task and procedure remained the same as in Experiment 1. An example of the stimuli is depicted in Figure 3.

The single-target condition was omitted in Experiment 2 [similarly as in the previous two-stream RSVP studies by Śmigasiewicz et al. (2010) and Verleger et al. (2009, 2010, 2011)], because to test the RH overload hypothesis we only needed to investigate whether the normally observed LVF advantage in T2 identification under the dual-target condition will reverse to a RVF advantage.

### Results

**Figure 4:**
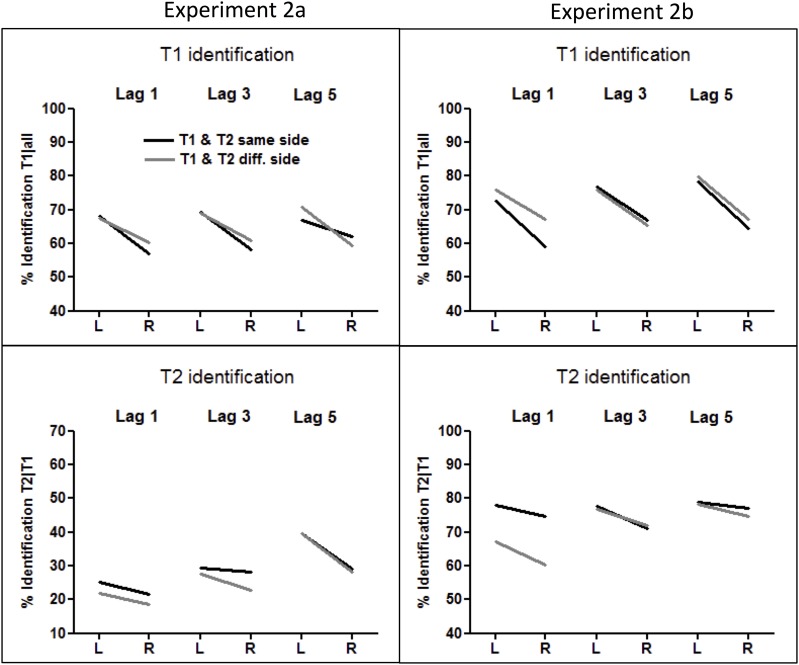
**Rates of T1 and T2 identification in Experiments 2A (left panel) and 2B (right panel).** T1 identification rates were calculated from all trials, and T2 identification rates were computed from all correctly identified T1 trials.

Mean identification rates of T1 and T2 are presented in Table 2 and on the left side of Figure 4.

**Table 2:** **Percentages of correct target identification (with standard deviations) in Experiments 2A, 2B, (with faces) and 3 (with shapes)**.

**Lag**	**1**				**3**				**5**			
**Side of the other target**	**Same**		**Opposite**		**Same**		**Opposite**		**Same**		**Opposite**	
**Target VF**	**Left**	**Right**	**Left**	**Right**	**Left**	**Right**	**Left**	**Right**	**Left**	**Right**	**Left**	**Right**
**T1**												
Experiment 2A	68.1 (12.2)	56.9 (10.5)	67.6 (13.4)	60.2 (14.0)	69.3 (13.1)	58.1 (13.0)	68.9 (13.6)	60.8 (12.5)	66.8 (13.8)	62.0 (12.2)	70.8 (13.3)	59.4 (13.6)
Experiment 2B	72.7 (11.4)	59.2 (10.8)	76.0 (14.0)	67.2 (10.1)	76.9 (14.4)	67.0 (14.3)	76.0 (12.7)	65.3 (12.8)	78.3 (10.8)	64.6 (12.2)	79.7 (10.7)	67.2 (12.4)
Experiment 3	82.4 (11.1)	87.7 (9.9)	82.3 (12.2)	86.0 (8.5)	79.8 (13.5)	83.2 (11.7)	82.2 (10.7)	85.5 (9.5)	79.7 (13.5)	87.0 (9.2)	80.1 (11.4)	86.8 (9.0)
**T2/T1 DUAL TARGET TASK**												
Experiment 2A	25.1 (13.7)	21.7 (12.9)	21.9 (10.5)	18.6 (7.8)	29.4 (14.5)	28.2 (14.7)	27.6 (11.8)	22.7 (12.8)	39.5 (22.0)	29.2 (17.9)	39.5 (16.9)	28.2 (9.9)
Experiment 2B	78.1 (16.8)	74.7 (16.2)	67.2 (11.1)	60.2 (13.6)	77.7 (12.5)	71.1 (16.7)	76.9 (14.1)	72.1 (13.4)	79.0 (14.0)	77.0 (15.1)	78.3 (15.4)	74.6 (14.3)
Experiment 3	55.3 (30.3)	51.5 (27.0)	33.3 (16.3)	20.2 (13.0)	48.0 (22.4)	39.6 (21.4)	36.7 (23.0)	28.9 (19.2)	52.6 (25.4)	37.4 (23.1)	48.5 (27.3)	37.4 (22.5)

#### T1 identification

T1 was correctly identified in 64% of trials (thus significantly worse than letter-T1 in Experiment 1, *F*_(1, 41)_ = 153.5, *p* < 0.0001, for the comparison to the procedure with standard stimuli), and 9% better in the LVF than in the RVF (*F*_(1, 20)_ = 9.4, *p* = 0.006, η^2^ = 0.32). When T1 occurred on the same side as the following T2, this LVF advantage was not significant at lag 5 (*F*_(1, 20)_ = 1.8, *p* = 0.18, η^2^ = 0.09; interaction T1 side × Lag for T1 occurring at the same side as T2: *F*_(2, 40)_ = 3.8, *p* = 0.041, η^2^ = 0.16; three-way interaction T1 Side × Side Change × Lag: *F*_(2, 40)_ = 5.6, *p* = 0.011, η^2^ = 0.22). In the opposite-side condition, the LVF advantage equaled 9% and did not differ between lags (*F*_(2, 40)_ = 1.3, *p* = 0.27, η^2^ = 0.06).

#### T2 identification

The T2-face was identified in only 28% of T1-correct trials (less than the digit-T2 in Experiment 1, *F*_(1, 41)_ = 229.5, *p* < 0.0001). Crucially, T2 was still identified significantly better in the LVF than in the RVF (6% difference, *F*_(1, 20)_ = 19.7, *p* < 0.0001, η^2^ = 0.49). The LVF advantage marginally increased at lag 5 (T2 Side × Lag: *F*_(2, 40)_ = 3.0, *p* = 0.066, η^2^ = 0.13). None of the other interactions with T2 Side was significant.

### Discussion

A clear LVF advantage was observed in identification of face-T2, in stark contrast to the RVF advantage predicted by the overload hypothesis. Already face-T1 was identified much better in the LVF. This is consistent with RH dominance in face processing (Dien, 2009). On the other hand, the fact that the LVF advantage already occurred with T1 might reflect the increased difficulty of this task, such that the RH dominance in attentional selection becomes apparent already with T1. This increased task difficulty by using faces instead of letters and digits severely compromised overall task performance, reducing accuracy in identification of both T1 and T2, as compared to the standard procedure with alphanumerical stimuli. Faces might be too similar to each other, thereby being much harder to distinguish than letters or numbers. Such similarity of stimuli would provide much greater burden for working memory than in case of letters or digits, and may also somehow confuse participants, which together increased the number of errors.

## Experiment 2B

Because the procedure applied in the previous experiment proved to be very difficult, we conducted an additional experiment, attempting to increase overall accuracy by reducing the sets of T1 and T2 from six to only two stimuli. Although this change from six to two targets may entail increasing the number of lucky guesses, from 17 to 50%, it allows for avoiding confusions due to high similarity between targets and the resulting overly high demands on memory. The expected higher accuracy should provide more reliable evidence in favor or against the overload hypothesis.

### Method

#### Participants

Participants were recruited from the same population and fulfilled the same inclusion criteria as in Experiment 1. The sixteen participants were 1 man and 15 women, their mean age was 20.0 (*SD* = 1.4), and their scores in the Edinburgh Handedness Inventory were 83.7 (*SD* = 17.3).

#### Stimuli, apparatus, and procedure

The sizes of the T1 and T2 sets were reduced from six to two. The number of distractor faces remained the same. The responses were matched to the requirement to select one of two alternatives only. Participants responded by pressing the “F” or “J” keys for T1 identification and the “1” or “4” keys for T2 identification, indicated by the response screens that followed the RSVP series in each trial like in the preceding experiments. All other parameters of stimuli, apparatus, and procedure remained the same as in Experiment 2A.

### Results

Mean identification rates of T1 and T2 are presented in Table 2 and on the right side of Figure 4.

#### T1 identification

T1 was correctly identified in 71% of trials. Similar to Experiment 2A, we observed a clear LVF advantage, which equaled 11% (*F*_(1, 15)_ = 12.3, *p* = 0.003, η^2^ = 0.45) and did not interact with other factors.

#### T2 identification

The overall identification rate amounted to 74%. Crucially, left T2 was identified 5% better than right T2 (*F*_(1, 15)_ = 10.2, *p* = 0.006, η^2^ = 0.40). No interaction of this effect with other factors was significant.

### Discussion

Replacing alphanumeric stimuli by faces, and in particular the red-letter T1 by a face on red background and the digit-T2 by a female-face T2, again resulted in LVF advantage in both T1 and T2 identification. Although the asymmetry was smaller than the effect obtained with the standard alphanumeric stimuli in Experiment 1, the direction of the effect provides clear evidence against the overload hypothesis, which predicted a LVF/RH disadvantage. The smaller effect size of the LVF advantage, as compared to the standard procedure, might be related to the different type of stimuli or to the larger difficulty of target discrimination, which resulted in lower accuracy.

## Experiment 3

In order not to prematurely reject the overload hypothesis on the basis of one particular type of stimuli only, the third experiment was conducted with another type of stimuli that is supposed to be processed preferentially by the RH. In particular, processes of coding and distinguishing between global and configural properties of objects or shapes have been shown to be right-lateralized (Gazzaniga, 2000; Floel et al., 2004; Hellige et al., 2010). Hence, in order to engage the RH more than the LH and to decrease the engagement of the left verbal system to a minimum, irregular geometric shapes were here used as T1 and as distractor stimuli. The shapes were all new, designed for the purpose of the study, thus were unknown to participants, and unnamable or at least very difficult to name, especially when displayed rapidly in serial presentation. Choosing this kind of non-verbal stimuli should prevent participants from providing verbal or analytic coding (cf. Hellige et al., 1979). As T2, a hexagon with a gap on one of its sides was used, in order to create a category of stimuli that would be relatively similar to the other shapes, but at the same time noticeably distinguishable, like digits among letters. According to the overload hypothesis, the pattern of VF asymmetry will be reversed from the standard procedure, i.e., a RVF advantage is expected. Alternatively, if the LVF advantage occurs due to RH dominance, the general pattern of asymmetry would remain principally unchanged.

### Method

#### Participants

Twenty-one participants took part in the experiment. Two of them had to be excluded due to high error rates in identifying T1 (32% correct, the average from the other 19 participants being 83%). In the remaining sample 15 were female. The average age was 20.0 (*SD* = 1.4), with average Edinburgh Handedness Inventory scores of 81.7 (*SD* = 22.0).

#### Stimuli, apparatus, and procedure

**Figure 5:**
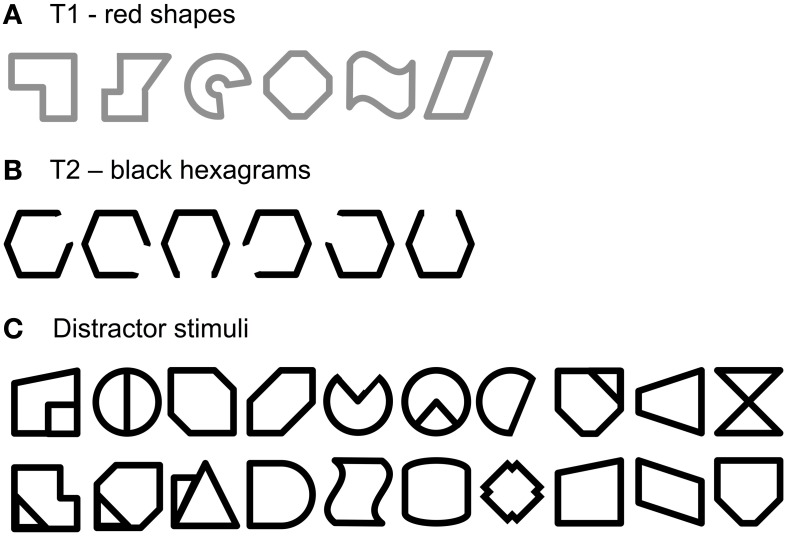
**Stimuli used in Experiment 3 as T1 (A), T2 (B), and distractors (C).** Red color is replaced here by gray.

The RSVP task used in this experiment was a faithful copy of the task from Experiment 2A, but irregular shapes were presented instead of faces. The stimuli are illustrated in Figure 5. Shapes were designed especially for the purpose of the study. All of them were irregular, unknown, and rather difficult to name or verbalize. The distractor set consisted of 20 black shapes. The set of T1 consisted of six red shapes, similar to, but different in details from distractors. The set of T2 included six hexagons with a gap: each hexagon had one of its six sides removed. As before, participants were asked to identify T1 and T2. At the end of each trial the response screen displayed the six targets and the participants were to press the corresponding key on the computer keyboard. As in Experiments 1 and 2A, the mapping of T1 and T2 to keys was 'D', 'F', 'G', 'J', 'K', and 'L' for T1s, and from '1' to '6' for T2s.

### Results

**Figure 6:**
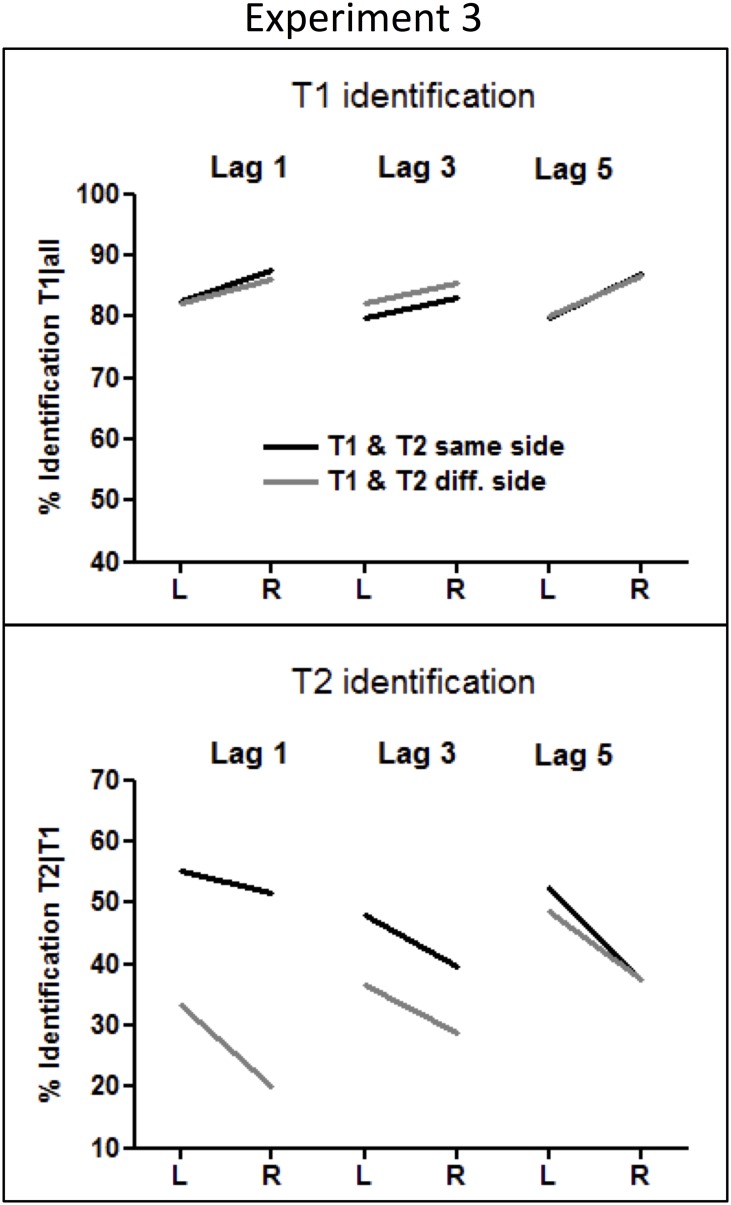
**Rates of T1 and T2 identification in Experiment 3.** T1 identification rates were calculated from all trials, and T2 identification rates were computed from all correctly identified T1 trials.

Mean identification rates of T1 and T2 are presented in Table 2 and in Figure 6.

#### T1 identification

Participants correctly identified shape-T1 in 83% of trials. Surprisingly, shape-T1s were identified about 5% better in the RVF than in the LVF (*F*_(1, 18)_ = 18.4, *p* < 0.0001, η^2^ = 0.50). Other effects of T1 Side were not significant.

#### T2 identification

The overall accuracy in hexagram-T2 identification was 41%. A clear LVF advantage, amounting to 10%, was obtained (*F*_(1, 18)_ = 22.6, *p* < 0.0001, η^2^ = 0.55). As indicated by the interaction of T2 Side × Side Change × Lag, *F*_(2, 36)_ = 4.3, *p* =.024, η^2^ = 0.19, this LVF advantage increased across lags when T1 and T2 occurred on the same side (T2 Side × Lag: *F*_(2, 36)_ = 6.1, *p* = 0.007), from the marginally significant 4% effect at lag 1 (*F*_(1, 18)_ = 3.3, *p* = 0.087), to 8% at lag 3 (*F*_(1, 18)_ = 7.0, *p* = 0.016), and to 15% at lag 5 (*F*_(1, 18)_ = 15.8, *p* = 0.001), and a stable LVF advantage of 11% across lags was observed when T1 and T2 occurred on different sides (T2 Side × Lag: *F* < 1.0).

### Discussion

The overall pattern of T2 identification rates across all conditions was very similar to the results obtained in the standard two-stream RSVP task with letters and digits. In particular, a clear LVF advantage in T2 identification was observed despite replacing all alphanumeric stimuli by shapes, and the digit-T2 by a hexagon-T2. Therefore, the results seem to disprove once again the overload hypothesis and suggest that the LVF advantage in T2 identification is independent of the type of stimuli used.

However, the red-shape T1 was identified better in the RVF than in the LVF. One possible explanation of this effect is that the requirement to keep the particular six types of red-shape T1 in mind overloaded visual working memory of the RH to such an extent that a reversed VF asymmetry was already produced in T1 identification, analogously to paradoxical effects of LVF advantage observed in a visuo-spatial verbal task when the LH was overloaded by a concurrent verbal memory task (Hellige et al., 1979). This account would, therefore, concede that overload is a potent factor in these RSVP tasks. However, since the overload account of the LVF advantage of T2 identification requires the LH to be overloaded, rather than the RH, this overload of the RH, although presumably present, cannot account for the obtained result.

A nearby alternative account of the better identification of T1 in the RVF is that this identification might require some specific processing capability in which the LH is more efficient, just as letters are supposed to do (cf. the slight RVF advantage for T1 in Experiment 1). For example, distinguishing between the specific T1 exemplars might require processing of spatial details rather than processing the global form. Processing of spatial details might be better accomplished by the LH (e.g., Robertson and Lamb, 1991) or some subset of the T1 shapes might have been verbally coded by participants. In this case, in terms of the overload hypothesis, it would be the LH that was overloaded by its successful identification of T1, reducing its ability to identify T2. Thus, if this alternative is true the current experiment did not disprove the overload hypothesis.

## General discussion

When two consecutive targets, T1 and T2, are presented in two simultaneous RSVP streams, T2 is identified much better in the LVF than in the RVF (Holländer et al., 2005; Verleger et al., 2009). According to the overload hypothesis (Hellige et al., 1979; Verleger et al., 2010), this asymmetry might reflect a LH disadvantage due to an overload of the LH's verbal processing system by the two-stream rapid serial presentation of distractor and target letters. If this hypothesis holds true, the LVF advantage should largely decrease or even be completely eliminated when the number of letter-distractors is drastically reduced or when the letter-T1 does not have to be identified. Analogously, replacing letters and digits by stimuli which the RH is supposed to be specialized for (e.g., faces or irregular shapes) should overload the RH, thereby decrease its processing efficiency and lead to LVF disadvantage in T2 identification.

The results of the present study provide evidence on this issue. Most convincing evidence against the overload hypothesis was provided by Experiment 2, where a LVF advantage was obtained for face T2 stimuli embedded among face distractors and following face T1 stimuli, in the same direction as was obtained in Experiment 1, and in previous studies, for digit T2 embedded among letters and following letter T1 stimuli. Importantly, this LVF advantage for face-T2 in Experiment 2 occurred in spite of a distinct LVF advantage already for face-T1. This face-T1 asymmetry, suggesting an advantage of the RH in identifying these faces, was even more marked than the reversed asymmetry (with a slight RVF advantage) obtained with the letter-T1 in Experiment 1. Thus, the overload hypothesis predicts that T2 asymmetry should be reversed, from a LVF advantage with alphanumeric stimuli to a RVF advantage with faces. This prediction was not borne out. Converging, though not unambiguous, evidence was provided by the other two experiments. First, the LVF advantage in T2 identification remained, despite decreased verbal load by reducing the number of background letter distractors (Experiment 1). Second, replacing letters and digits by irregular shapes and hexagrams (Experiment 3) did not reverse the asymmetry. The LVF advantage was still present with those stimuli, contrary to the predictions of the overload hypothesis. Thus, the study provides evidence that this asymmetry is largely independent from the verbal load level (Experiment 1), from the type of stimuli used as both targets and distractors (Experiments 2 and 3), as well as from the presence and direction of VF asymmetry in T1 identification (Experiment 3).

In support of the overload hypothesis, it may be argued for Experiment 1 (cf. 2.3, above) that the reduction of number of distractors in Experiment 1 was not sufficiently drastic, with even four preceding stimuli (in case of lag 1) perhaps being enough to overload the LH. Moreover, waiving the requirement to identify T1 (Experiment 1) reduced the LVF, which conforms to the overload hypothesis. Correspondingly, for Experiment 3 (cf. 5.3, above) the RVF advantage for identifying the shape-T1 puts into doubt whether these shapes were as specifically processed in the RH as we would have expected them to be (and as the faces in Experiment 2 probably were). Thus, evidence is still not conclusive, in spite of the wide variation in stimuli used in our experiments.

Yet, the most parsimonious explanation of the constantly occurring LVF advantage in T2 identification is that the effect is brought about by lateralization of some domain-general processing system, plausibly an attentional or perceptual mechanism, as has been hypothesized in previous studies (Holländer et al., 2005; Verleger et al., 2009, 2011; Śmigasiewicz et al., 2010). Evidence that conforms to both the attentional and the perceptual explanations of the LVF advantage was obtained in recent two-stream RSVP studies by recording two components of event-related electroencephalogram potentials (ERPs) which were the N2pc evoked by T1 and T2, and the visual evoked potentials (VEPs) triggered by the stream of distractors. N2pc is defined as a negative deflection recorded above the visual cortex contralateral to attended stimuli, as compared with responses to irrelevant non-target or unattended stimuli, and is interpreted as an indicator of attentional selection (Luck et al., 1993; Eimer, 1996; Wascher and Wauschkuhn, 1996). Shorter latencies of the T2-evoked N2pc were obtained in the RH than in the LH, suggesting RH superiority in speed of T2 selection (Verleger et al., 2009, 2011). Furthermore, the visual potentials evoked by the distractor streams preceding T1 were reliably leading at the RH by a few milliseconds compared to the LH (Verleger et al., 2011, 2013), which suggests generally faster perceptual processing of visual events in the RH than in the LH in this task (cf. Okon-Singer et al., 2011). This general speed advantage of the RH might contribute to the efficiency of the RH in singling out the rapidly presented target-stimuli within the two streams of distractors.

The attentional explanation appears to be in line with the neuroanatomical model of attentional selection proposed by Corbetta and Shulman (2002). Those authors have provided many pieces of evidence for distinguishing between two neural systems dedicated for attentional selection: the dorsal frontoparietal network controlling endogenous orienting of attention, which is driven by expectations or predictive cues, and the ventral frontoparietal network controlling selection of targets or other potentially relevant stimuli that occur outside of the current focus of attention (see Corbetta et al., 2008; Shulman and Corbetta, 2012 for review). The latter system is strongly lateralized, with the temporo-parietal junction in the right hemisphere constituting one of its crucial neural nodes, whereas the dorsal network is organized bilaterally, including the intraparietal sulcus and the frontal eye field of both hemispheres. The lateralized organization of the ventral attentional network conforms to behavioral results showing LVF advantages in selection of unattended targets (Evert et al., 2003; Asanowicz et al., 2012). In the two-stream RSVP task, participants do not know exactly where and when targets will occur, thus constant monitoring of both streams is needed for successfully selecting T2, providing a typical situation of competitive processing (Desimone and Duncan, 1995). In such case, the ventral attentional system would have to be constantly engaged during performing the task (cf. Shulman and Corbetta, 2012). Because this right-lateralized network has direct access to the information from the LVF, this information may be favored in this competition, whereas RVF information has yet to be relayed through the corpus callosum, which takes more time and also may somewhat degrade the relayed percept, as would be predicted from the callosal relay model of functional lateralization (Zaidel, 1983; Moscovitch, 1986). If this scenario holds true, then the LVF advantage in T2 identification should be a function of the degree of involvement of the ventral orienting system, which might be manipulated by cueing of T2 location (cf. Shulman et al., 2010). The system is supposed to be least involved after valid cues, because then T2 would be presented directly to the focus of attention directed by the cue, moderately involved in some neutral-cue condition, and most involved after invalid cues, because then T2 would be presented at uncued location while attention is focused on the cued location (cf. Corbetta and Shulman, 2002). Recent behavioral and ERP experiments from our laboratories seem to confirm this prediction, showing the expected gradient of asymmetry across the three cue conditions in the two-stream RSVP task (in preparation).

The perceptual explanation is, on the other hand, in line with the notion of the RH's greater efficiency in initial, early visuospatial processing (Hellige and Webster, 1979; Grabowska and Nowicka, 1996). Several studies have shown that an LVF/RH advantage was observed when stimuli were perceptually degraded by manipulating parameters like exposure duration, retinal eccentricity, luminance, contrast, and blurring, even in tasks in which the LH is supposed to be dominant and, accordingly, a RVF advantage is usually observed (see Christman, 1989; Grabowska and Nowicka, 1996 for review). More direct evidence for this hypothesis was provided by an ERP study showing higher amplitude of N1 and P2 VEP components in the right than in the left hemisphere, recorded from occipital regions during processing of very briefly presented (30 ms) grating stimuli (Grabowska et al., 1992). The two-stream RSVP task seems to entail a rather extreme case of visibility degradation, greatly increasing demands for the perceptual system, because stimuli in this task are presented very rapidly, with short exposure duration, with retinal eccentricity, and simultaneously in both VFs. Thus, behavioral results from the two-stream RSVP task showing the LVF advantage, as well as the above-mentioned asymmetry in latency of early VEPs (Verleger et al., 2011, 2013) may be seen as conforming to the perceptual hypothesis.

## Conclusion

The present two-stream RSVP experiments have shown that the LVF advantage in identifying rapidly presented target stimuli is neither appreciably decreased by reducing the hypothesized overload of the LH nor reversed into a RVF advantage by attempting to overload the RH. Thereby, although not entirely disproving the overload hypothesis, these results suggest as the most parsimonious explanation that the asymmetry may be related to RH superiority, plausibly both in initial perceptual processing and in attentional selection.

### Conflict of interest statement

The authors declare that the research was conducted in the absence of any commercial or financial relationships that could be construed as a potential conflict of interest.

## Footnotes

^1^This shows that the asymmetry cannot be explained by uncontrolled eye movements. Another argument (pointed out by one reviewer of this paper) is that if the LVF advantage were eye movements artifacts, the asymmetry should be consistent for both T1 and T2, which was not the case in any of the two stream RSVP studies conducted thus far.
